# From Axonal Growth to Neurodegeneration: The Dual Role of Neurofilament Dynamics in Health and Disease

**DOI:** 10.3390/neurosci7030058

**Published:** 2026-05-09

**Authors:** Yikang An, Hongying Lan, Jialong Xiong, Ruoyan Jing, Dongjin Gu, Haoyang Zhang, Xinping Liu, Qi Zhao, Feng Wang

**Affiliations:** 1 School of Life Science, Beijing Institute of Technology, Beijing 100081, China; 17352276126@163.com (Y.A.); 18810507276@163.com (H.L.); xjl000305@163.com (J.X.); 3220232014@bit.edu.cn (R.J.); wingo753232@163.com (D.G.); 3120241427@bit.edu.cn (H.Z.); m18137986508@163.com (X.L.); 2 Cancer Centre, Institute of Translational Medicine, Faculty of Health Sciences, University of Macau, Taipa, Macau SAR 999078, China; 3 MoE Frontiers Science Center for Precision Oncology, University of Macau, Taipa, Macau SAR 999078, China

**Keywords:** neurofilaments, intermediate filaments, axonal growth, assembly mechanisms, neurodegenerative diseases, expression switching, amyotrophic lateral sclerosis, Charcot–Marie–Tooth disease, protein aggregation, aberrant phosphorylation

## Abstract

Neurofilaments (NFs) are the predominant type IV intermediate filaments in differentiated neurons, functioning not just as static scaffolds, but as active drivers of radial axonal growth and nerve conduction velocity. While their physical properties are well characterized, a critical gap remains in synthesizing how their dynamic assembly and developmental subunit switching directly dictate neurodegenerative outcomes. This review breaks down the molecular architecture and stepwise kinetic assembly of NFs, detailing their role in polarized transport and the formation of a protective viscoelastic gel network within axons. We specifically highlight the physiological expression switching of early subunits, such as alpha-internexin and peripherin, during neuronal maturation, a process often overlooked in traditional structural reviews. By examining how specific gene mutations and aberrant hyperphosphorylation trigger axonal transport jams and protein aggregation, we map the direct pathways leading to amyotrophic lateral sclerosis (ALS) and Charcot–Marie–Tooth (CMT) disease. Finally, we emphasize that a precise mechanistic decoding of NF structural dynamics and their pathological disruption is essential for understanding the fundamental etiology of these neurodegenerative conditions.

## 1. Introduction

Neurons represent the most morphologically complex and polarized cells within the human body, distinguished primarily by their dendritic networks for signal reception and axons for long distance information transmission [1]. In humans, the axons of certain motor neurons can exceed one meter in length [2]. The maintenance of such extreme morphological dimensions and the execution of extensive intracellular transport rely upon an organized and robust internal cytoskeleton system [3,4]. Comprising predominantly microtubules, microfilaments or actin filaments, and intermediate filaments (IFs), the cytoskeleton operates through a dynamic three-dimensional network to cooperatively sustain the structural integrity and functional polarity of neurons [5,6].

Within mature, particularly large myelinated nerve fibers, neurofilaments constitute the most abundant cytoskeletal component in axons [7], often outnumbering microtubules by a factor of several to dozens [8]. Belonging to the type IV intermediate filament proteins encoded by a multigene family [9], the primary biological significance of neurofilaments lies in providing structural mechanical support within neurons [10]. Exhibiting mechanical flexibility and tensile strength [11], NFs offer robust mechanical reinforcement to the elongated and fragile axons, effectively preventing physical rupture or damage during daily physiological movements and tissue stretching [12].

Beyond functioning as a static physical scaffold, the dynamic physiological relevance of NFs is underscored by their direct role in dictating radial axonal growth [3]. According to the cable theory in neurophysiology, the cross-sectional area of an axon is inversely proportional to its internal longitudinal resistance, which in turn determines the conduction velocity of action potentials [13]. Concomitant with neuronal development and myelination, NFs continuously accumulate and expand outward within the axon, driven by the mutual repulsion of their long and negatively charged C-terminal tails, thereby forcing a significant increase in axonal caliber [14]. Consequently, NFs serve not only as the skeletal framework maintaining cellular morphology but also as the essential material basis guaranteeing efficient and rapid network communication across the entire central and peripheral nervous systems [15].

Precisely because NFs hold a central position in sustaining neuronal survival and conduction functions, any aberrations in their assembly, transport, or chemical modifications, such as hyperphosphorylation, can trigger devastating consequences [16].

Substantial evidence indicates that mutations in neurofilament genes or the subsequent formation of abnormal protein aggregates constitute pathological factors in various neurodegenerative diseases, including Charcot–Marie–Tooth disease (CMT) and amyotrophic lateral sclerosis (ALS) [17,18].

Moving beyond the traditional view of neurofilaments as merely static structural supports, this review deconstructs their unique molecular architecture and stepwise kinetic assembly. We specifically address a critical yet frequently overlooked dimension: the dynamic expression switching of early intermediate filaments (α-internexin and peripherin) during axonal maturation. By bridging these fundamental biological mechanisms with pathological states, we delineate how specific gene mutations and aberrant phosphorylation trigger axonal transport collapse in diseases such as ALS and CMT. Through comprehensively integrating these molecular defects with the fundamental principles of NF assembly and transport, we aim to provide a systematic framework for understanding how cytoskeletal dysregulation drives neuronal degeneration.

## 2. Materials and Methods

We performed a narrative literature search on neurofilament dynamics in axonal growth and neurodegeneration. Articles were identified through PubMed, Web of Science, and Scopus databases, covering publications from neurofilament discovery to December 2025. Search terms included “neurofilament”, “axonal growth”, “axonal transport”, “neurodegeneration”, “ALS”, “Charcot-Marie-Tooth”, “NEFL”, “NEFM”, “NEFH”, “peripherin”, “alpha-internexin”, “phosphorylation”, “assembly”, and “biomarkers”.

Inclusion criteria encompassed studies on neurofilament structure, assembly mechanisms, developmental expression, axonal transport, disease-associated mutations, and biomarker applications. Exclusion criteria comprised studies unrelated to neurofilament function or lacking sufficient methodological details. Articles were selected based on relevance to understanding neurofilament roles in physiological growth and pathological neurodegeneration, including molecular studies, in vitro/in vivo experiments, human genetic studies, and clinical investigations.

Data were extracted on subunit composition, assembly kinetics, phosphorylation states, transport velocities, mutation types, disease phenotypes, biomarker levels, and therapeutic approaches. Information was synthesized to emphasize key molecular pathways, pathogenic mechanisms, and translational implications. This narrative approach was selected due to heterogeneity in study designs and methodologies across the literature.

## 3. Results

### 3.1. Molecular Structure of Neurofilaments

**Figure 1:**
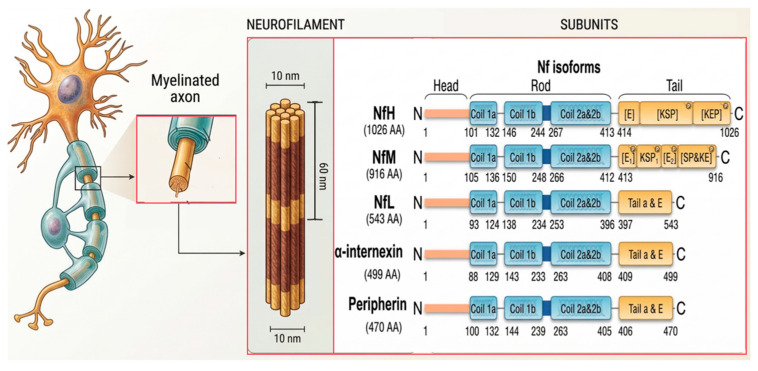
Molecular structure of neurofilament proteins. Domain Architecture: Illustrates the five primary type IV intermediate filament subunits, including the neurofilament triplet of NF-L, NF-M, and NF-H, alongside alpha-internexin and peripherin which are expressed in specific neurons. All subunits share the conserved tripartite topological configuration comprising the head, rod and tail. The N-terminal head orchestrates the initiation of polymerization; the central rod marked in blue, constituted by conserved alpha-helical segments, serves as the core for inter-subunit intertwining; the C-terminal tails vary in length, with NF-L, alpha-internexin, and peripherin possessing shorter tails, while NF-M and NF-H extend exceptionally massive long tails that harbor KSP repeats capable of undergoing high-density multiple phosphorylations. “P” stands for phosphorylation site.

Neurofilaments within mature mammalian axons constitute a complex heteropolymeric structure [19]. The subunits comprising this network belong predominantly to the type IV intermediate filament (IF) proteins. In addition to the classical triplet consisting of neurofilament light (NF-L), medium (NF-M), and heavy (NF-H) chains, the mature network also incorporates alpha-internexin (a type IV IF predominantly found in the central nervous system) and peripherin (a type III IF predominantly found in the peripheral nervous system), which are expressed and co-assembled at specific sites and developmental stages [20]. As shown in Figure 1, despite differences in molecular weight and biochemical properties, all five subunits retain the conserved tripartite topology of the intermediate filament protein family, consisting of a head, a rod domain, and a tail [21].

N-terminal head domain: This is a sequence-variable and flexible region, whose amino acid composition is enriched in serine and threonine residues [9]. Acting as the catalytic substrate for specific protein kinases, the dynamic phosphorylation modifications of the head domain directly function as a spatial conformational switch that regulates subunit association and initiates polymerization [22].

Central rod domain: This represents the most evolutionarily conserved physical backbone of the entire subunit, composed of continuous alpha-helical segments containing regular hydrophobic heptad repeats [9]. It is the interaction of these hydrophobic surfaces that directly drives the coiled-coil intertwining between two adjacent subunits [23].

C-terminal tail domain: The tails of NF-L, INA, and PRPH are relatively short and remain largely concealed within the filament core; conversely, NF-M and NF-H extend long tail sequences enriched with negatively charged glutamic acid and classical KSP phosphorylation repeat motifs [24]. Upon completion of assembly, these long tails breach the core confines, extending outward radially, and dictate the physical spacing between filaments via electrostatic repulsion [25].

### 3.2. Dynamic Assembly Process of Neurofilaments

The assembly of neurofilaments follows the generalized multi-step kinetic principles of the intermediate filament family, yet it is dependent in vivo on NF-L, INA, or PRPH serving as the core backbone template:

Dimer Formation: The initial step of assembly commences at the central rod domains of two subunits, which must include a backbone subunit. Through molecular recognition of their hydrophobic heptad repeats, they intertwine in a parallel and in-register manner to form a stable coiled-coil heterodimer or homodimer.

Tetramer Association: Subsequently, two dimers associate laterally in an antiparallel and half-staggered fashion to form a tetramer. Because this antiparallel arrangement mutually cancels out the molecular head-to-tail polarity, the resulting tetramer and subsequent neurofilaments are devoid of the plus-minus polarity characteristic of directed transport seen in microtubules.

Unit-Length Filament (ULF): After losing polarity, approximately eight tetramers undergo rapid lateral aggregation to form a short cylindrical complex measuring about 16 nm in diameter and 60 nm in length, termed the unit-length filament (ULF), which acts as the foundational building block in assembly kinetics.

**Figure 2:**
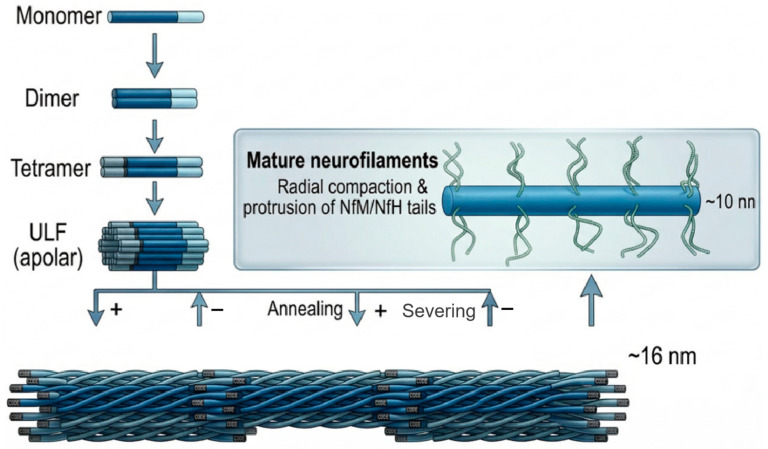
Schematic model of neurofilament protein assembly. Both the in vitro and in vivo assembly of neurofilaments follow strict stepwise kinetics: Dimer formation: The rod domains of monomers coil in parallel and in-register via hydrophobic interactions to form dimers. Tetramer formation: Two dimers associate in an antiparallel, half-staggered arrangement. Unit-length filament (ULF): Approximately eight tetramers aggregate laterally in rapid succession to form short, cylindrical ULFs with a diameter of about 16 nm and a length of about 60 nm. Elongation and Compaction: ULFs are joined end-to-end longitudinally via annealing and, following radial compaction, form mature neurofilaments roughly 10 nm in diameter.

Longitudinal Elongation and Radial Compaction: Finally, ULFs connect end-to-end longitudinally via annealing to rapidly elongate into nascent filaments spanning several micrometers. Immediately thereafter, the filament interior undergoes structural rearrangement and radial compaction, shrinking to a mature neurofilament with a diameter of approximately 10 nm. At this stage, the modified long tails of NF-M and NF-H protrude fully outward from the compacted structural core, forming the characteristic cross-bridge structures visible under electron microscopy that sustain the three-dimensional axonal network [26,27,28,29], as shown in Figure 2.

### 3.3. Expression Switching of Neurofilaments

Throughout the development and differentiation of neurons, the protein composition of neurofilaments is far from static. The five neurofilament subtypes continuously alter their expression patterns in accordance with neuronal maturation, a physiological process designated as neurofilament expression switching.

#### 3.3.1. Predominant Components of Early Neurofilaments

During the initial stages of neuronal development, such as axonal sprouting and early elongation, cells demand a cytoskeletal network that possesses high dynamicity, structural flexibility, and the capability for rapid polymerization. At this time, early intermediate filament proteins, specifically the type IV alpha-internexin distributed mainly in the central nervous system and the type III peripherin in the peripheral nervous system, exhibit high expression levels. By virtue of their robust homopolymeric capacities, these early subunits can rapidly assemble into filaments, thereby delivering initial mechanical scaffolding and structural guidance for nascent and dynamic neurites [30].

#### 3.3.2. Expression Switching and Component Substitution During Maturation

As axons successfully project to their target tissues and initiate the myelination process, their core physiological mandate transitions from longitudinal elongation to radial thickening. Because the C-terminal tails of alpha-internexin and peripherin are relatively short and lack the sidearm structures required to generate spatial steric hindrance and electrostatic repulsion, they cannot satisfy the physical demands of luminal expansion in mature axons. Accordingly, neurons execute an expression switch at the level of gene regulation, upregulating the transcription of NF-M and NF-H subunits equipped with ultra-long tails to drive the mature expansion of the axon [31].

#### 3.3.3. Secondary Roles and Spatial Relocation in Adult Tissues

**Figure 3:**
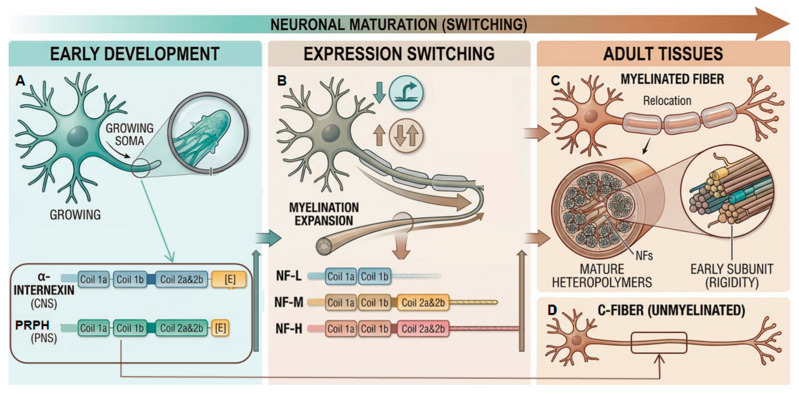
Schematic of Neurofilament Expression Switching. The diagram illustrates dynamic changes in neurofilament (NF) subtype expression patterns across neuronal development, maturation, and adult tissues. Early Development (**A**): Predominant homopolymeric expression of α-internexin (CNS) and peripherin (PNS), forming short-tailed, dynamic filament structures that provide mechanical guidance and support. Maturation Switching (**B**): Gene regulation drives a switch to upregulation of NF-M and NF-H subunits with ultra-long tails, driving axoplasm expansion via steric hindrance and electrostatic repulsion. The onset of myelination is a key trigger. Adult Tissues: Early subunits do not vanish but are relocated. They persist primarily in two forms: 1. Co-assembling into the classical heteropolymeric network at low stoichiometries as a fourth subunit (**C**); 2. Persisting independently in thin, unmyelinated fibers like C-fibers (**D**).

Early traditional perspectives once postulated that alpha-internexin and peripherin would completely cease expression and vanish from the cytoskeletal network once neuronal cells achieved developmental maturity. However, subsequent investigations have confirmed that these proteins are never entirely eliminated. To optimize the allocation of cellular synthetic resources while sustaining the mechanical rigidity of the mature axonal skeleton, the expression levels of these two early intermediate filaments are downregulated during maturation, accompanied by a shift in their spatial localization. In adult individuals, they persist primarily in two forms; on the one hand, they bind directly at extremely low stoichiometric ratios as the fourth subunit, co-assembling with the classical neurofilament triplet into a mature heteropolymeric network [32], and on the other hand, their independent expression becomes strictly confined to thin, unmyelinated nerve fibers, such as C-fibers, where they continue to uphold the structural foundation of these specific neurons [33], as shown in Figure 3.

### 3.4. Physiological Functions and Kinetic Mechanisms of Neurofilaments

Neurofilaments form a dynamic, cross-linked three-dimensional network within neurons. Their principal physiological functions—and the molecular and biophysical mechanisms that support them—can be conceptualized across several core dimensions. Beyond their well-established structural roles in axons, emerging evidence indicates that neurofilaments also localize to postsynaptic terminals. Specific neurofilament subunits interact directly with synaptic vesicle proteins and NMDA-type glutamate receptors, and these interactions modulate synaptic plasticity, influence neurotransmission, and ultimately shape cognitive behavior [34].

#### 3.4.1. Polarized Directional Transport and Spatial Accumulation

Since mature nerve axons inherently lack the ribosomal machinery required for synthesizing structural proteins, all neurofilament subunits must be synthesized within the cell body and initially assembled into short filament precursors [35,36]. Incapable of autonomous motility, these short fibers must attach to molecular motors, such as kinesin or dynein, and migrate towards the nerve terminals utilizing microtubules as tracks [37]. As the organism develops and neurons mature, the microtubule density within the axon decreases significantly, which likely causes the NFs continuously transported from the cell body to accumulate in the middle and distal segments of the axon, thereby laying the material foundation for subsequent axonal expansion [38,39].

#### 3.4.2. Electrostatic Repulsion and Radial Axonal Expansion

The retained NFs align longitudinally and in parallel within the narrow axonal conduit; at this juncture, the unique and negatively charged C-terminal sidearms of the neurofilament heavy (NF-H) and medium (NF-M) chains begin to exert critical effects [40]. Electrostatic repulsion occurs between the sidearms of adjacent filaments, and this space-filling effect and repulsive force radially distend the originally slender nerve axon in all directions [14]. Dictated by the cable theory of neurophysiology, the cross-sectional area of the expanded axon increases markedly, resulting in a reduction in its internal longitudinal resistance [41]. Thus, NF-driven radial expansion not only optimizes cellular morphology but also serves as the core physical prerequisite guaranteeing the intracellular conduction of action potentials [42]. Furthermore, in vivo studies utilizing knockout mouse models have unequivocally confirmed these structural roles. The targeted deletion of either NF-M or NF-H directly impairs radial growth. Specifically, mice lacking the NF-M subunit exhibit significant reductions in myelinated axonal caliber, demonstrating its absolute requirement for establishing proper interfilament spacing [43,44].

#### 3.4.3. Viscoelastic Gel Network and Mechanical Shock Absorption

Beyond generating repulsive forces, the sidearms of NFs undergo dynamic cross-linking with neighboring NFs, microtubules, and microfilaments, forming a viscoelastic gel network within the axon that exhibits properties akin to a non-Newtonian fluid [45,46]. Concurrently, these sidearms tightly entwine and anchor the transporting microtubule tracks as well as organelles like mitochondria [47]. This specialized physical phase state endows the axon with resilience against tensile and compressive forces [12]. Upon external impact to the organism’s head or severe physical stretching of nerve tissues, this elastic gel network woven by NF sidearms becomes taut, dissipating localized stress throughout the entire axonal lumen and thereby absorbing mechanical shock to an extent to prevent neuronal rupture [48]. In studies utilizing animal models with specific NF gene knockouts, mice congenitally lacking the NF network experience extensive axonal severing even upon sustaining mild traumatic brain injury [49].

#### 3.4.4. Dynamic Equilibrium of Axonal Transport and Length-Dependent Regulation

Once axonal development is complete and its caliber stabilized, the internal NFs do not remain static but rather enter a complex dynamic equilibrium [50]. In mature nerve fibers, over 80 percent of NFs possess the capacity for mobility [51]. This unique mechanism permits long filaments to constantly fine-tune their positions within the conduit, thereby maintaining the uniformity of their overall distribution [52]. NFs rarely travel at a constant velocity; they spend the majority of their time in a stationary state, transitioning into rapid shuttling movements once mobilized.

However, the transport efficiency of these filaments exhibits length dependency: Short filaments under 10 micrometers in length demonstrate high directional persistence with relatively unidirectional trajectories, rarely engaging in frequent back-and-forth movements. Moreover, short filaments are susceptible to end-to-end joining during transport, splicing into longer filaments via annealing, an event with a remarkably high probability of 1.24 occurrences per hour. Long filaments exceeding 20 micrometers in length encounter significantly heightened net resistance against their movement as they grow. Research indicates that for every 1-micrometer increment in length, the net transport velocity of long filaments drops by 0.016 µm/s, pause durations increase by 1.1 percent, and directional persistence decreases by 1.5 percent, noting that the decline in net velocity stems from frequent oscillating movements rather than a reduction in absolute speed per transit. More critically, ultra-long filaments are prone to inducing axonal traffic jams, which is why they face a very high probability of being actively severed, reaching up to 1.5 times per hour.

**Figure 4:**
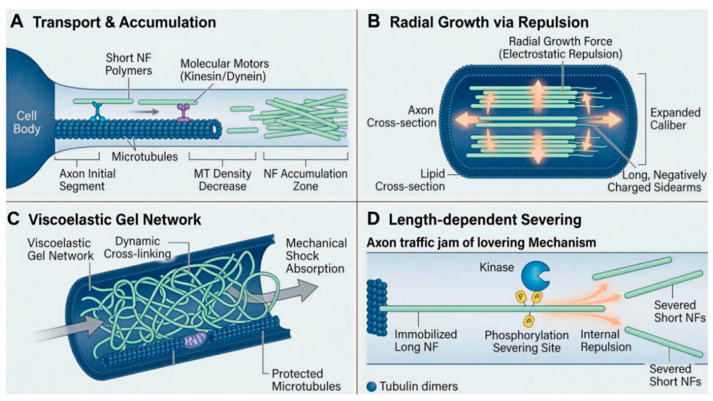
Schematic representation of the core physiological functions and dynamic mechanical regulatory mechanisms of neurofilaments (NFs) within the axon. (**A**) Polarized directional transport and spatial accumulation. Short neurofilament precursors, fully assembled within the cell body, attach to molecular motors such as kinesin or dynein and undergo polarized transport toward the distal axon using microtubules as tracks. As the axon elongates, microtubule density decreases significantly, leading to extensive retention and accumulation of neurofilaments within the lumen. (**B**) Radial axonal expansion driven by electrostatic repulsion. In the longitudinal section of the axon, the retained and parallel aligned neurofilaments generate intense mutual electrostatic repulsive forces, indicated by orange arrows, via their characteristic negatively charged long C-terminal sidearms. This spatial repulsive effect drives the radial expansion of the axon, ly increasing its cross-sectional area and lowering longitudinal resistance. (**C**) Viscoelastic gel network and mechanical shock absorption. The sidearms of neurofilaments undergo dynamic cross-linking with microtubules and other cytoskeletal components, forming a viscoelastic gel network characterized by non-Newtonian fluid properties. This network physically anchors organelles such as mitochondria and, when the axon is subjected to external mechanical stretching or impact, rapidly dissipates the applied stress through network tension, effectively safeguarding the nerve fiber from physical rupture. (**D**) Length-dependent phosphorylation and severing mechanism. When ultra-long neurofilaments stall in the transport network or cause traffic congestion, specific kinases catalyze the addition of negatively charged phosphate groups to their core regions. The resulting strong internal electrostatic repulsion shatters the long filament from the middle, releasing numerous maneuverable short filament fragments to maintain the dynamic equilibrium and fluidity of axonal transport.

Kinase-mediated phosphorylation and severing mechanism: When a long filament becomes trapped at a roadblock on the transport track for extended periods, the cell activates a rescue mechanism. Specific kinases catalyze the conjugation of strongly negatively charged phosphate groups to the inter-subunit head regions located in the middle of the long filament. The ensuing electrostatic repulsion disrupts inter-subunit interactions, snapping the long filament into several shorter segments from its center. Upon being severed and shortened, these filaments regain high maneuverability and rapidly disperse, thereby ensuring the fluidity and vitality of the entire axonal transport network [53], as shown in Figure 4.

#### 3.4.5. Controversies in Axonal Transport

The physical state of transported neurofilaments remains debated. The subunit transport model suggests that neurofilaments move as individual monomers or unpolymerized unit-length filaments (ULFs). They assemble after reaching their destination. The polymer transport model suggests that neurofilaments move as fully assembled polymers. Recent studies propose a hybrid model. Mature polymers form the primary cargo. A small pool of soluble subunits exists for repair [54]. Recognizing these competing models is necessary. Therapeutic strategies must target the specific form of the transport blockage.

### 3.5. The Role of Neurofilament Mutations and Post-Translational Modifications in Neurological Diseases

Pathologically relevant mutations in neurofilament genes and their aberrant post-translational modifications, particularly phosphorylation, play a role in the onset and progression of diverse neurodegenerative diseases. Recent systematic studies have not only expanded the traditional map of pathogenic mutations but also illuminated the crucial roles of synonymous and protective variants in determining genetic susceptibility to disease.

#### 3.5.1. Neurofilament Gene Mutations: Mendelian Disorders and Genetic Susceptibility

The stability of the neurofilament network relies on the precise assembly of its subunits. Genetic variations across NF genes exhibit distinct disease specificities, manifesting as Mendelian disorders or as risk-modifying factors in neurodegenerative diseases.

Charcot–Marie–Tooth Disease (CMT): Mutations in the NEFL gene constitute a genetic cause of CMT type 2E, an autosomal dominant Mendelian disorder. In an epidemiological study involving a Japanese cohort, approximately 9 percent of CMT patients harbored NEFL mutations [55]. Mechanistically, most NEFL missense mutations (such as Pro22Ser) occur within the N-terminal head or central rod domains. These mutations disrupt the ability of NF-L to co-assemble into unit-length filaments (ULFs), leading to axonal transport collapse and peripheral neuropathy. By contrast, CMT induced by NEFH mutations is rare (incidence of 0.003 percent in a UK cohort) and displays a specific domain distribution, clustering within the C-terminal tail [56]. These tail frameshift variants produce aberrant polypeptides that alter electrostatic repulsion and the cross-linking network, resulting in axonal constriction.

Amyotrophic Lateral Sclerosis (ALS): Unlike the Mendelian inheritance seen in CMT, most NF gene variants associated with ALS (found in NEFL, NEFM, NEFH, and PRPH) act as genetic susceptibility factors that increase disease risk [57]. For example, mutations in the PRPH gene alter the assembly dynamics of early intermediate filaments, predisposing motor neurons to form inclusion bodies under stress. Genetic variants are not exclusively pathogenic; certain variations demonstrate protective effects. For instance, an intronic variant within the NEFH gene (rs140814097) reduces the risk of spinal-onset ALS by 50 percent [58,59].

Cross-Associations in Other Degenerative Diseases: The spectrum of neurofilament mutations extends to other conditions. An NEFH mutation, P1007A, has been reported in cases of spinal muscular atrophy (SMA) [60]. Furthermore, variations in NEFM (e.g., S336G) and INA (e.g., E46K) are considered genetic susceptibility factors for Parkinson’s disease (PD) and Lewy body dementia (LBD) [61,62]. These variants alter cytoskeletal flexibility and protein clearance mechanisms, contributing to neurodegeneration in aging neurons.

#### 3.5.2. Physiological Mechanisms and Pathological Impacts of Post-Translational Modifications

Neurofilaments undergo extensive post-translational modifications (PTMs), with NF-H being one of the most phosphorylated proteins in the human body. These modifications, primarily occurring on serine (Ser), threonine (Thr), and tyrosine (Tyr) residues, significantly influence protein charge, molecular weight, and structural dynamics [63].

Physiological Regulation and Axonal Development: A distinct division of labor exists among kinases. The C-terminal tail of NF-H undergoes high-density catalysis mediated by proline-directed kinases [60], whereas the N-terminal head is regulated by non-proline-directed kinases (e.g., CDK5, GSK-3) [64]. Physiologically, this phosphorylation-induced electrostatic repulsion serves as the core mechanism for enlarging and maintaining axonal diameter [65]. It regulates the dynamic remodeling of the cytoskeleton [66], bolsters resistance to proteolytic degradation [67], and is delicately modulated by myelin-associated glycoprotein (MAG) during postnatal development [68,69].

Pathological Disruption and PTM Crosstalk: The disruption of this PTM homeostasis is a fundamental driver of neurodegenerative pathology [70]. Researchers have mapped specific targets, such as Ser54 on NF-H and Ser23 on NF-M, furnishing a molecular foundation for comprehending aberrant aggregation [71]. In states like ALS and AD, the physiological “topographic phosphorylation” pattern is severely compromised [22]. Hyperphosphorylation of the N-terminal head domains directly inhibits the assembly of unit-length filaments (ULFs) and promotes the disassembly of existing filaments into neurotoxic oligomers [70].

Crucially, this pathogenic cascade is amplified by the cross-talk between different PTMs. For instance, a reduction in O-GlcNAcylation levels on NF-M and NF-H leaves adjacent residues vulnerable to hyperphosphorylation and subsequent degradation [72,73]. Additionally, under conditions of chronic oxidative stress, peroxynitrite-mediated nitration targets specific tyrosine residues, particularly within the head and rod domains of NF-L. This modification disrupts intersubunit contacts, inhibiting the normal assembly of neurofilaments and promoting the cytoskeletal pathology strongly associated with ALS [74,75]. Collectively, these aberrant modifications shatter the dynamic equilibrium of axonal transport, converting a functional scaffold into a physical blockade [70]. Despite these mechanistic insights, the temporal hierarchy of pathological events remains unresolved.

Distinguishing Correlation and Causation: The literature documents hyperphosphorylation in ALS and AD. It is necessary to distinguish correlation from causation. A debate exists regarding the sequence of events. Aberrant kinase activation may act as the primary trigger that dismantles the network. Alternatively, hyperphosphorylation may be a secondary consequence of prior transport stalling [76]. Determining the exact sequence requires further in vivo studies. This distinction affects the development of kinase-inhibitor therapeutics, as shown in Table 1.

### 3.6. Neurofilaments as Translational Biomarkers of Axonal Injury

The structural specialization of neurofilaments makes them ideal indicators of neuronal integrity. Upon axonal membrane disruption or severe proteolytic cleavage during neurodegeneration, NF subunits—most notably the soluble neurofilament light chain (NfL)—are released from the axoplasm into the interstitial fluid, subsequently circulating into the cerebrospinal fluid (CSF) and peripheral blood [77]. This transition from an intracellular structural scaffold to an extracellular signaling marker represents a fundamental shift in our ability to monitor neuroaxonal health in vivo.

Currently, the advent of ultrasensitive assays, such as Single Molecule Array (Simoa) technology, has enabled the reliable detection of trace NfL in serum or plasma, transforming it into a accessible, “universal” biomarker for neuroaxonal damage [78]. Unlike traditional biomarkers, NfL levels correlate directly with the intensity of axonal degeneration across a spectrum of disorders. In ALS, blood NfL concentrations correlate strongly with the rate of functional decline and can elevate months before clinical symptoms manifest, offering a critical window for early intervention [79]. Similarly, phosphorylated neurofilament heavy chain (pNfH) has emerged as a complementary prognostic marker, particularly in acute neurodegenerative states where its robust resistance to proteolysis provides a stable readout of large-caliber axonal loss [64].

Despite its high sensitivity, the “universal” nature of neurofilaments as biomarkers also presents challenges in diagnostic specificity, as elevated levels reflect generalized axonal injury rather than a specific disease etiology. Future research focusing on the ratio of different NF subunits or the integration of NF dynamics with other neuroimaging modalities will be essential to enhance the precision of these molecular tools. By monitoring the pathological “leakage” of these structural components, clinicians can gain a dynamic window into the otherwise hidden process of axonal degeneration, successfully bridging the gap between basic cytoskeletal biology and clinical neurology [77,78], as shown in Table 2.

## 4. Discussion

Functioning as a specialized and dynamic cytoskeletal system intrinsic to differentiated neurons, neurofilaments fulfill the biophysical prerequisites for efficient neural conduction through their sophisticated structural specialization and tightly regulated expression programs. Throughout neuronal maturation, the five neurofilament subtypes undergo coordinated shifts in expression, a process that not only provides spatiotemporally precise mechanical support for axonal growth but also highlights the intrinsic plasticity of the cytoskeletal network.

Despite these advances, fundamental research in this field continues to face major bottlenecks. Authentic neurofilament heteropolymers still lack atomic-level structural resolution, leaving current models of native assembly mechanisms dependent on extrapolations from homologous intermediate filament proteins. Moreover, the dynamic interaction networks between neurofilaments and other cellular components—such as microtubules and mitochondria—together with their intricate regulatory processes, remain incompletely understood. Pathologically, neurofilament abnormalities have been firmly implicated as causal contributors to both peripheral and central neurodegenerative disorders. Yet, although the clinical consequences of cytoskeletal disruption are well documented, the initiating molecular events that drive aberrant neurofilament aggregation remain insufficiently defined.

Beyond these molecular uncertainties, the interpretation of neurofilament behavior in vivo is further complicated by biological and translational variables. Age exerts a profound influence on neurofilament dynamics: the viscoelastic network becomes increasingly rigid with advancing age, baseline axonal transport velocities decline, and the propensity for protein aggregation rises. Neuronal subtype adds another layer of complexity. Large-caliber spinal motor neurons, enriched in NF-H, are disproportionately vulnerable to transport perturbations—consistent with the selective motor neuron loss characteristic of ALS—whereas small unmyelinated interneurons are typically spared. Sex also modulates neurofilament biology. Clinical studies indicate that males generally exhibit higher baseline circulating NfL concentrations than females, and sex-specific hormonal environments may influence disease trajectories, underscoring the need for sex-stratified reference intervals in future clinical applications.

Translating preclinical findings to human pathology introduces additional challenges. Many earlier studies merged data from murine models, cell lines, and human tissues despite fundamental anatomical and biophysical differences. Human motor neurons possess exceptionally long axons, imposing transport demands that rodent neurons cannot fully replicate. Although rodent models remain valuable for elucidating basic assembly kinetics, their translational validity for late-stage inclusion body formation is inherently limited.

## 5. Conclusions

Future research endeavors must pivot from phenomenological descriptions toward mechanistic deciphering of pathology. Primarily, there is an urgent imperative to uncover how aberrant post-translational modifications, induced by the dysregulation of specific kinases, shatter the dynamic equilibrium of axonal transport and instigate protein aggregation. Secondly, attaining atomic-resolution structural elucidations of NF heteropolymers is critical for clarifying their native assembly mechanisms. Ultimately, integrating high-resolution structural biology with dynamic functional assays will furnish a breakthrough for deciphering the precise etiology of neurodegenerative disorders driven by cyto-skeletal failure.

## Figures and Tables

**Table 1:** Genetic loci, molecular weights, and disease associations of neurofilament subunits.

	NfH	NfM	NfL	INA	PRPH
Chromosome	22	8	8	10	12
Gene location	29,480,218–29,491,390	24,913,761–24,919,093	24,950,955–24,956,612	49,295,147–49,298,686	49,295,147–49,298,686
Length 1 ^a^	1020	916	543	499	470
Length 2 ^b^	924	540	–	–	–
Weight 1 (Mw) ^c^	112,477.56734 ± 7.23404	102,470.81258 ± 6.54664	61,400.80804 ± 3.95853	55,389.99135 ± 3.54851	53,650.26292 ± 3.46054
Weight 2 (kDa) ^d^	105.6	102.5	61.5	55.4	53.7
Weight 3 (kDa) ^e^	190–210	150	68	66	57
Charge ^f^	−11	−64	−49	−14	−15
Phosphorylation	+++ ^g^	++	+	+	+
O-glycosylation	++	++	+	–	–
Genetic risk for	ALS/SMA, CMT	ALS, PD	ALS, CMT	PD, LBD	ALS

Notes. The weight increases following translation due to post-translational modifications, the most important of which is phosphorylation. A number of mutations have been associated with an increased risk for disease which is either autosomal dominant, autosomal recessive or considered a genetic susceptibility factor. Da stands for Dalton; Mw indicates molecular weight. “^a^” denotes full protein length; “^b^” indicates shorter lengths following alternative splicing; “^c^” means calculated from DNA sequence; “^d^” stands for reported from processed DNA sequence; “^e^” represents migration in SDS gel which differs from the calculated weights because of post-translational modification; “^f^” signifies calculated from amino acid sequence; “^g^” highlights that NfH is the most extensively phosphorylated protein of the human body.

**Table 2:** Neurofilament Levels Across Major Neurological Diseases: Biofluid Sources, Clinical Applications, and Relative Changes.

Disease State	Biofluid	Clinical Utility/Application	Relative Level Compared with Healthy Controls
Amyotrophic Lateral Sclerosis (ALS)	Blood/CSF	Prognostic indicator; monitoring disease progression; presymptomatic detection	Markedly elevated (commonly several-fold higher; increases with disease stage)
Charcot–Marie–Tooth Disease (CMT)	Blood	Marker of axonal loss and disease severity, particularly in axonal subtypes (e.g., CMT2E)	Moderately to markedly elevated
Alzheimer’s Disease (AD)	Blood/CSF	Tracking longitudinal neurodegeneration; correlates with cognitive decline	Moderately elevated
Multiple Sclerosis (MS)	Blood	Monitoring inflammatory activity; assessing response to disease-modifying therapies	Moderately elevated, fluctuating with relapse activity

## Data Availability

No new data were created or analyzed in this study. Data sharing is not applicable to this article.
